# γ1 GABA_A_ Receptors in Spinal Nociceptive Circuits

**DOI:** 10.1523/JNEUROSCI.0591-24.2024

**Published:** 2024-08-13

**Authors:** Elena Neumann, Teresa Cramer, Mario A. Acuña, Louis Scheurer, Camilla Beccarini, Bernhard Luscher, Hendrik Wildner, Hanns Ulrich Zeilhofer

**Affiliations:** ^1^Institute of Pharmacology and Toxicology, University of Zurich, CH-8057 Zurich, Switzerland; ^2^Departments of Biology, Biochemistry and Molecular Biology, and Psychiatry and Penn State Neuroscience Institute, Pennsylvania State University, University Park, Pennsylvania 16802; ^3^Institute of Pharmaceutical Sciences, Swiss Federal Institute of Technology (ETH) Zurich, CH-8093 Zurich, Switzerland

**Keywords:** receptor clustering, dorsal horn, GABA_A_ receptor subtype, gephyrin, nociception, pain

## Abstract

GABAergic neurons and GABA_A_ receptors (GABA_A_Rs) are critical elements of almost all neuronal circuits. Most GABA_A_Rs of the CNS are heteropentameric ion channels composed of two α, two β, and one γ subunits. These receptors serve as important drug targets for benzodiazepine (BDZ) site agonists, which potentiate the action of GABA at GABA_A_Rs. Most GABA_A_R classifications rely on the heterogeneity of the α subunit (α1–α6) included in the receptor complex. Heterogeneity of the γ subunits (γ1–γ3), which mediate synaptic clustering of GABA_A_Rs and contribute, together with α subunits, to the benzodiazepine (BDZ) binding site, has gained less attention, mainly because γ2 subunits greatly outnumber the other γ subunits in most brain regions. Here, we have investigated a potential role of non-γ2 GABA_A_Rs in neural circuits of the spinal dorsal horn, a key site of nociceptive processing. Female and male mice were studied. We demonstrate that besides γ2 subunits, γ1 subunits are significantly expressed in the spinal dorsal horn, especially in its superficial layers. Unlike global γ2 subunit deletion, which is lethal, spinal cord-specific loss of γ2 subunits was well tolerated. GABA_A_R clustering in the superficial dorsal horn remained largely unaffected and antihyperalgesic actions of HZ-166, a nonsedative BDZ site agonist, were partially retained. Our results thus suggest that the superficial dorsal horn harbors functionally relevant amounts of γ1 subunits that support the synaptic clustering of GABA_A_Rs in this site. They further suggest that γ1 containing GABA_A_Rs contribute to the spinal control of nociceptive information flow.

## Significance Statement

Our results identify for the first time a CNS area (the spinal dorsal horn) in which atypical GABA_A_ receptors containing the γ1 subunit serve a physiological role in the synaptic clustering of GABA_A_ receptors. They also show that pharmacological modulation of γ1 GABA_A_ receptors by a nonsedative GABA_A_ receptor modulator alleviates chronic pain in neuropathic mice.

## Introduction

GABAergic neurons and GABA_A_Rs are essential elements of most if not all CNS circuits. Endogenous or drug-induced changes in the GABAergic tone have profound effects on mental states, including, among others, wakefulness, sleep, and anxiety, and various behaviors, such as pain and itch related reactions. Most GABA_A_Rs are heteropentameric proteins that contain two α, two β, and one γ subunits (Sieghart and Sperk, 2002). While α and β subunits jointly form the GABA binding sites, the anchoring of GABA_A_Rs to postsynaptic membranes depends on the γ subunit and the postsynaptic scaffold protein gephyrin (Essrich et al., 1998). Together with an α subunit, the γ subunit is in addition an essential part of the high affinity benzodiazepine (BDZ) binding site of GABA_A_Rs (Ernst et al., 2003).

Most BDZs potentiate the action of GABA at GABA_A_Rs that contain an α1, α2, α3, or α5 subunit together with a γ2 subunit. Much work has been done to attribute specific pharmacological actions of BDZs to GABA_A_R subtypes defined by the α subunit included in the receptor complex. For instance, the sedative and anxiolytic actions of benzodiazepines have respectively been attributed to GABA_A_Rs containing α1 or α2 subunits (Rudolph et al., 1999; Low et al., 2000; McKernan et al., 2000). At the spinal level, GABA_A_Rs containing α2 and α3 subunits (α2 and α3 GABA_A_Rs) control the relay of nociceptive (pain related) and pruritoceptive (itch related) information (Knabl et al., 2008; Ralvenius et al., 2015, 2018). Compounds that target α2 or α3 GABA_A_Rs, but not α1 GABA_A_Rs, have been developed in a quest for nonsedative anxiolytics (Atack, 2009; Rudolph and Knoflach, 2011), and subsequent work has shown that such compounds also exert antihyperalgesic and antipruritic effects in different animal models (Knabl et al., 2008; Di Lio et al., 2011; Ralvenius et al., 2018; Neumann et al., 2021).

Much less is known about the contribution of γ subunit diversity to the functional heterogeneity of GABA_A_Rs. Most CNS GABA_A_Rs contain a γ2 subunit (Gunther et al., 1995) and most actions of BDZ site agonists occur through γ2 GABA_A_Rs (Gunther et al., 1995; Ernst et al., 2003). However, γ1 and γ3 GABA_A_Rs may still be expressed at biologically relevant quantities in certain CNS areas and may serve important functions. One such CNS area might be the spinal dorsal horn. Previous work has suggested that *Gabrg1* is relatively densely expressed in its superficial layers, the so-called substantia gelatinosa (www.gensat.org/imagenavigator.jsp?imageID=12994). At this site, nociceptive nerve fibers enter the CNS and form synapses with projection neurons and local excitatory and inhibitory interneurons. Diminished GABAergic inhibition leads to exaggerated pain sensations and a shift in perception from pleasant touch to pain (Beyer et al., 1985; Yaksh, 1989; Sivilotti and Woolf, 1994; Foster et al., 2015). Potentiation of GABAergic inhibition at this site is antihyperalgesic in different animal models of inflammatory and neuropathic pain (Knabl et al., 2008, 2009; Di Lio et al., 2011; Braz et al., 2012; Reichl et al., 2012; Ralvenius et al., 2015; Neumann et al., 2021).

In the present study, we have analyzed the expression of the three GABA_A_R γ subunits in the mouse spinal dorsal horn and investigated morphological, behavioral, and pharmacological phenotypes of mice lacking the γ2 GABA_A_R subunit from the spinal cord. Our results show that besides γ2, γ1 is significantly expressed in the spinal cord, especially in the superficial layers, where it is coexpressed in the same neurons with α2 and α3 subunits. In contrast, γ3 was expressed only in very low amounts. Unlike global γ2 subunit deletion, which is lethal (Gunther et al., 1995), the spinal cord-specific loss of γ2 did not lead to obvious deficits. The clustering of GABA_A_Rs in the superficial dorsal horn was only mildly affected, and antihyperalgesic actions of the nonsedative BDZ site agonist HZ-166 were partially retained suggesting that γ1 GABA_A_Rs contribute to GABA_A_R clustering and BDZ site agonist-mediated potentiation of spinal GABA_A_Rs.

## Materials and Methods

### Mice

Experiments were performed in wild-type mice, in mice that lack the γ2 subunit specifically from the spinal cord (*hoxB8-γ2*^−/−^ mice) and in *γ2^fl/fl^* littermates. *HoxB8-γ2*^−/−^ mice were generated by crossing *γ2*^fl/fl^ mice (Schweizer et al., 2003) with *hoxB8-cre* mice (Witschi et al., 2010), which allow brain-sparing conditional gene deletion. All mouse lines were maintained on a C57BL/6J background.

Permission for animal experiments was obtained from the Veterinäramt des Kantons Zürich (231/2017) prior to the start of the experiments. During all experiments, we closely adhered to the ARRIVE guidelines and the UK Animals (Scientific Procedures) Act, 1986, and associated guidelines, EU Directive 2010/63/EU for animal experiments.

### Drug and drug administration

HZ-166 [8-ethynyl-6-(2-pyridinyl)-4H-imidazo[1,5-a][1,4]benzodiazepine-3-carboxylic acid ethyl ester; Cook et al., 2006; Rivas et al., 2009] was kindly provided by Dr. James Cook, Milwaukee Institute for Drug Discovery, University of Wisconsin Milwaukee. TPA023B [6,2′-difluoro-5′-[3-(1-hydroxy-1-methylethyl)imidazo[1,2-*b*][1,2,4]triazin-7-yl]biphenyl-2-carbonitrile; Compound 11, in Russell et al. (2006)] was obtained from PharmaBlock Sciences (Nanjing). For intrathecal (i.t.) injections, HZ-166 was suspended in artificial cerebrospinal fluid (aCSF) containing (in mM) 120 NaCl, 5 HEPES, 26 NaHCO_3_, 1.25 NaH_2_PO_4_, 2.5 KCl, 2 CaCl_2_, 1 MgCl_2_, and 10 glucose, pH 7.35. Intrathecal injections were performed under isoflurane (1.5%) anesthesia with a 30 Gauge stainless steel needle (Thermo Fisher Scientific) as reported previously (Neumann et al., 2021). For per oral (p.o.) administration, TPA023B was suspended in 0.9% saline and 1% Tween 80 and a metal (stainless steel) gavage needle (20 Gauge) was used (for details, see Neumann et al., 2021).

### Quantitative reverse transcriptase PCR

Lumbar dorsal root ganglia (DRGs) and spinal cords were rapidly removed from naive C57BL/6 mice of different age [embryonic day (E) 15 to postnatal day (P) 50] and from adult C57BL/6 mice 7 d after a chronic constriction injury (CCI) surgery of the left sciatic nerve. mRNA expression of all three GABA_A_R γ subunit-encoding genes (*Gabrg1*, *Gabrg2*, *Gabrg3*) was assessed by quantitative reverse transcriptase PCR (qRT-PCR) using β-actin as reference gene.

### RNAscope fluorescent in situ hybridization

Multiplex FISH (mFISH) was performed using the Manual RNAscope Assay (Advanced Cell Diagnostics, RRID:SCR_012481) on fresh frozen tissue. Sections were mounted onto Superfrost Plus glass slides (Thermo Fisher Scientific) and stored at −80°C prior to use. mFISH was performed according to the Manual RNAscope Multiplex Fluorescent Reagent Kit V2 (323100) user manual. Probes were revealed with TSA Vivid Fluorophore Dyes (520, 570, or 650). Depending on the TSA Vivid Fluorophore used, different levels of background staining were observed. A total of 2.4 µm stacks were acquired on a Zeiss LSM800 Pascal confocal microscope using a 1.3 NA × 40 EC Plan-Neofluar oil-immersion objective. Quantification was performed using the cell counter plug in Fiji (RRID:SCR_002285). Cells with three or more fluorescent puncta within an area 2 µm larger than the nucleus of the respective cell were counted as positive for the given marker.

The following RNAscope probes were used: Mm-Gabra2-C2 (435011-C2), Mm-Gabra3-C3 (435021-C3), Mm-Gabrg1 (501401), Mm-Gabrg2-C2 (408051-C2), Mm-Gabrg2 (408051), Mm-Slc32a1 (vGAT) (319191-C2), Mm-Slc17a6 (vGluT2) (319171-C3), Mm- Olig2-C3 (447091-C3), Mm-Aif1-C2 (319141-C2), and Mm-GFAP-C3 (313211-C3).

### Immunohistochemistry

The localization of γ2, α2, and α3 GABA_A_R subunits as well as of gephyrin was studied in 40-µm-thick lumbar spinal cord sections obtained from three male adult *hoxb8-γ2*^−/−^ and *γ2*^fl/fl^ mice. Animals were deeply anaesthetized with pentobarbital (Nembutal, 50 mg/kg, i.p.) and perfused with oxygenated aCSF. Spinal cords were rapidly collected, placed in ice-cold 4% PFA for 90 min, and cryoprotected overnight in a 30% sucrose/PBS solution. Subsequently, spinal cords were snap frozen with dry ice and cut in free-floating slices, kept in antifreeze at −20°C until the day of staining. GABA_A_R antibodies were home-made subunit-specific antisera raised in guinea pig (Fritschy and Mohler, 1995). Gephyrin was detected using the mouse monoclonal antibody mAb7a (Synaptic Systems, catalog #147021). Final dilutions were 1:10,000 (γ2), 1:1,000 (α2), 1:10,000 (α3), and 1:1,000 (gephyrin). For immunofluorescence staining, sections were incubated overnight at 4°C with a mixture of primary antibodies diluted in Tris buffer containing 2% normal goat serum. Sections were washed extensively and incubated for 1 h at room temperature with the corresponding secondary antibodies conjugated to Cy3 (1:500), Cy5 (1:200; Jackson ImmunoResearch), or Alexa 488 (1:1,000, Molecular Probes). Sections were washed again and coverslipped with fluorescence mounting medium (DAKO). Images of the labeled sections were acquired using a Zeiss LSM 800 microscope (Carl Zeiss) equipped with an 40× oil-immersion objective. All imaging parameters were kept constant between sections. A custom Python script using the ImageJ image-processing framework (openly available on a GitHub repository https://github.com/dcolam/Cluster-Analysis-Plugin) was used for puncta analysis. The plugin provides a rapid and unbiased puncta quantification tool in image analysis, as it allows the usage of both default and self-defined parameters. In brief, puncta identification using a default thresholding method and size cutoff of <0.2 and >3 μm in diameter was followed by the detection of their spatial overlap (colocalization). For colocalization, individual puncta detected were enlarged by 0.1 μm to prevent possible edge exclusions, and colocalization was defined when over 50% puncta overlapped. Representative example images were processed using ImageJ. Statistical tests were performed using Prism software (GraphPad).

### Electrophysiological analysis in HEK 293 cells

The effects of HZ-166 on currents through recombinant GABA_A_Rs were studied in HEK293 cells transiently expressing GABA_A_Rs. HEK293 cells were transfected using lipofectamine LTX. The transfection mixture contained (in μg) 1 α2/β3, 3 γ2, and 0.5 EGFP and 1 α2/β3, 3 γ1, and 0.5 EGFP (used as a marker of successful transfection). Whole-cell patch-clamp recordings of GABA-evoked currents were made at room temperature (20–24°C) 18–36 h after transfection. Cells were voltage clamped at −60 mV. The external solution contained (in mM) 150 NaCl; 10 KCl; 2.0 CaCl_2_; 1.0 MgCl_2_; 10 HEPES, pH 7.4; and 10 glucose. Recording electrodes were filled with internal solution containing (in mM) 120 CsCl; 10 EGTA; 10 HEPES, pH 7.40; 4 MgCl_2_; 0.5 GTP; and 2 ATP. GABA was applied to the recorded cell using a manually controlled pulse (4–6 s) of a low subsaturating and virtually nondesensitizing GABA concentration (EC_5_). GABA EC_5_ values were determined for α2β3γ*2* and α2β3γ1 GABA_A_Rs. EC_50_ values and Hill coefficients (*n*_h_) were obtained from fits of normalized concentration response curves to the equation *I*_GABA _= *I*_max_ [GABA]^*n*_h_^ / ([GABA]^*n*^_^h^ _+ [EC_50_]^*n*_h_^) using Igor Pro (WaveMetrics) software. *I*_max_ was determined as the average maximal current elicited by a concentration of 1 mM GABA. HZ-166 was dissolved in DMSO (final concentration <0.1%) and subsequently diluted with the recording solution to be coapplied together with GABA without preincubation.

### Chronic constriction injury surgery

Neuropathic pain was induced by applying a CCI (Bennett and Xie, 1988) to the left sciatic nerve proximal to the trifurcation with three loose (5-0, not absorbable) silk (Ethicon) ligatures. For that purpose, mice were anesthetized with isoflurane 1–3%. Afterward, skin was closed with 5-0 Dermalon sutures (Covidien).

### Behavioral tests

All behavioral experiments were performed in 7–10-week-old mice of either sex. Care was taken to ensure equal numbers of female and male mice in all groups. The female experimenter was blinded either to the genotype or the treatment with vehicle and drug.

Mechanical sensitivity was quantified as the change in the paw withdrawal threshold evoked by an electronic von Frey filament (IITC Life Science). Effects of HZ-166 on mechanical hyperalgesia were assessed 7 d after surgery using the electronic von Frey filament.

Percent maximal possible effect (%MPE) was calculated as follows:

$$\text{\%}\mathrm{MPE}(t)\;=\;(E(t)\;-\;E_{\mathrm{predrug}}))\;/\;(E_{\mathrm{preCCI}}\;-\;E_{\mathrm{predrug}}),$$

where MPE is the maximal possible effect; *E*(*t*) is the paw withdrawal thresholds at time point *t*; *E*_predrug_ is the *E* after CCI surgery but before HZ-166 application; and *E*_preCCI_ is the *E* baseline before CCI surgery.

Heat sensitivity was determined by the measurement of the hindpaw withdrawal latency to a defined radiant heat stimulus applied to the plantar surface of the left hindpaw, respectively. The latter experiments were performed using the Plantar Analgesia Meter (IITC Life Science) with the heat intensity set to 14. The floor plate was prewarmed to 37°C, and the cutoff time was set to 32 s to avoid tissue damage. Withdrawal latencies to noxious cold were assessed cooling the 5-mm-thick borosilicate glass platform directly under the mouse hindpaw using powdered dry ice compressed into a 1 cm large syringe (Brenner et al., 2015). Cold allodynia was measured as the time spent lifting, shaking, or licking the paw (s per min) after the application of acetone onto the affected paw.

Responses to light mechanical stimulation of the hairy skin were tested as the change in the paw withdrawal responses upon gentle stimulation with a paint brush. The following score was used: 0 (no evoked movement), 1 (walking away or brief paw lifting of 1 s or less), 2 (sustained lifting of >2 s), 3 (strong lateral lifting above a 90° angle), or 4 (flinching/licking of the affected paw). For the pin prick test, measurements were taken by stimulating the plantar surface of the mouse hindpaw with a blunted G26 needle. Six measurements were taken at an interval of 2 min and responses were scored as “0” for no reaction or “1” if the mouse responded.

Motor control was assessed on a rotarod instrument with the rod accelerating from 4 to 40 rpm within 5 min. Mice were placed on the rotarod and six measurements were taken per mouse. Muscle relaxation was measured using a metal horizontal that was placed 20 cm above the ground. Animals were assisted to place their forepaws on the wire. Successes and failures to grab the wire with at least one hindpaw were assessed.

Locomotor activity was assessed using an actimeter. Mice were placed into an area of 10 cm radius equipped with four pairs of light beams and photosensors. Locomotor activity was recorded for 120 min and analyzed between 60 and 120 min after TPA023B administration.

### Statistics

Unless otherwise noted, data are shown as mean ± SEM. When appropriate, data were analyzed using one-way ANOVA or two-way repeated-measures (ANOVA) or unpaired *t* tests followed by Bonferroni’s correction for multiple testing. Complete results of the statistical tests are provided in the figure legends. In all statistical analyses, results were considered significant if *p* < 0.05.

## Results

### Expression of GABA_A_R γ subunits in mouse DRG and spinal cord

**Figure 1.**
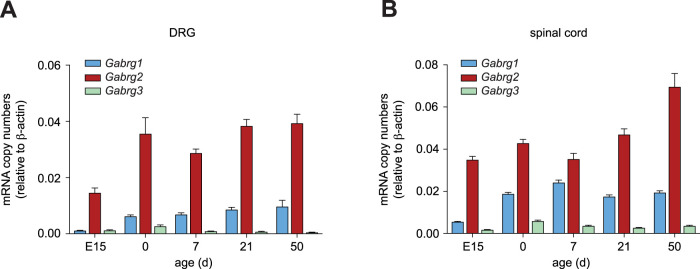
GABA_A_R γ subunit expression in mouse spinal cord and DRG. ***A***, qRT-PCR measurements of the three GABA_A_R γ subunits relative to β-actin in lumbar DRGs of naive mice. Developmental changes from E15 to adulthood (P50) (*n* = 5–7 mice). ***B***, Same as ***A*** but lumbar spinal cord.

We first used qRT-PCR to quantify the expression of GABA_A_R γ1, γ2, and γ3 subunits (encoded by *Gabrg1*, *Gabrg2*, and *Gabrg3* genes) in lumbar spinal cord tissue and DRG, which harbor the neurons that give rise to the peripheral sensory nerve fibers. We analyzed the expression at different developmental stages ranging from E15 to P50 (Fig. 1). *Gabrg2* was the most highly expressed GABA_A_R γ subunit gene during all developmental stages investigated both in DRG and spinal cord. In both tissues, *Gabrg1* expression was low at E15 but increased at birth and remained at relatively constant levels during postnatal development. Expression levels were ∼25 and 30% of those of *Gabrg2* in the DRG and spinal cord, respectively. Expression of the *Gabrg3* was generally very low with a small peak at P1 both in the DRG and spinal cord. These data show that mouse DRG and spinal cords contain not only high amounts of *Gabrg2* mRNA but also considerable amounts of *Gabrg1*, while *Gabrg3* mRNA is basically absent.

### Cellular distribution of γ1 and γ2 GABA_A_R in the mouse spinal cord

**Figure 2.**
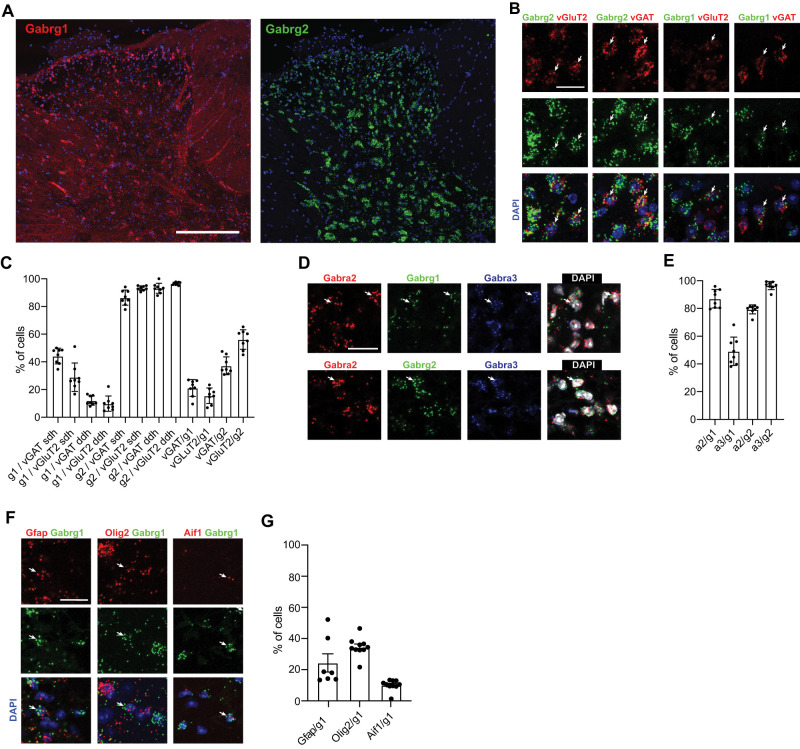
Cellular expression pattern of the GABA_A_R γ1 and γ2 subunits. ***A***, mFISH experiments showing expression of *Gabrg1* (left) and *Gabrg2* (right) in transverse lumbar spinal cord sections costained with DAPI to visualize cell nuclei. Scale bar, 200 µm. ***B***, Expression of *Gabrg1* and *Gabrg2* in glutamatergic (vGluT2) and GABAergic (vGAT) neurons in the superficial dorsal horn. Arrows indicate cells with coexpression. ***C***, Statistical analysis: g1, *Gabrg1*; g2, *Gabrg2*; sdh, superficial dorsal horn; ddh, deep dorsal horn. Mean ± SD. ***D***, Coexpression of *Gabrg1* and *Gabrg2* with *Gabra2* and *Gabra3*. Arrows indicate cells with coexpression. Scale bar, 20 µm. ***E***, Statistical analysis. g1, *Gabrg1*; g2, *Gabrg2*; a2, *Gabra2*; a3, *Gabra3*. Eight sections were analyzed per condition. Two sections were taken per mouse. Each dot represents a single section. ***F***, ***G***, Expression of *Gabrg1* in different types of non-neuronal cells. ***F***, mFISH using probers for *Gabrg1*, *GFAP* (astrocytes), *Olig2* (oligodendrocytes), and *Aif1* (microglia)*.* Arrows indicate cells coexpressing the respective marker with *Gabrg1*. Scale bar, 20 µm. ***G***, Statistical analysis. Mean ± SD.

We next investigated *Gabrg1* and *Gabrg2* expression on a cellular level in the lumbar spinal cord of adult (7 week old) mice (Fig. 2). On a gross scale, multiplex fluorescent in situ hybridization (mFISH) revealed that both *Gabrg1* and *Gabrg2* transcripts were found across the entire spinal dorsal horn (Fig. 2*A*). *Gabrg2* was present in virtually all GABAergic (vGAT positive) and glutamatergic (vGluT2 positive) neurons, both in the superficial and deep dorsal horn (Fig. 2*B*,*C*). The expression pattern of *Gabrg1* was less uniform. Within the superficial dorsal horn, *Gabrg1* was expressed in 44.2 ± 2.0% of GABAergic and in 29.0 ± 3.6% of glutamatergic neurons. Expression in the deep dorsal horn was lower, with 12.0 ± 1.2% of GABAergic and 9.8 ± 2.0% of glutamatergic neurons expressing *Gabrg1*. In total, 56.2 ± 2.5% of all *Gabrg2*-containing neurons were glutamatergic, while the remaining ones were GABAergic (37.2 ± 2.3%; Fig. 2*B*,*C*). *Gabrg1* was slightly more prevalent in GABAergic (21.2 ± 2.2%) than that in glutamatergic dorsal horn neurons (15.6 ± 2.0%), in line with the results of a previous single-cell RNA sequencing study (Haring et al., 2018).

We next analyzed whether GABA_A_R α subunits were coexpressed with *Gabrg1* and *Gabrg2* (Fig. 2*D*,*E*). Since GABA_A_R α2 and α3 subunits are the most prevalent α subunits in the spinal dorsal horn (Bohlhalter et al., 1996; Paul et al., 2012), we focused our analyses on these subunits. Most dorsal horn cells expressing *Gabrg1* also contained *Gabra2* and/or *Gabra3* (87.0 ± 2.4% and 49.1 ± 3.6% for *Gabra2* and *Gabra3*, respectively). Almost all *Gabrg2-*containing neurons (96.5 ± 1.0%) also contained *Gabra3* and 79.3 ± 1.2% contained *Gabra2* (Fig. 2*E*).

Interestingly, much of the *Gabrg1* expression (∼63%) associated with cell bodies (DAPI-positive structures) could not be localized to GABAergic or glutamatergic neurons (compare Fig. 2*C*), suggesting significant expression in non-neuronal cells. Since *Gabrg1* was often localized in thin elongated structures presumed axons (compare Fig. 2*A*), it seemed conceivable that the non-neuronal expression is in oligodendrocytes ensheathing neuronal axons, consistent with the report by Ordaz et al. (2021). Additional mFISH experiments (Fig. 2*F*,*G*) verified that *Gabrg1* was expressed in oligodendrocytes (*Olig2* positive cells), but also in astrocytes (*Gfap* positive cells) and microglia, identified by expression of the *Aif1* gene, which encodes for the microglia marker IBA1.

Taken together, these experiments confirm that *Gabrg1* is expressed both in inhibitory and excitatory neurons and in different glial cells of the dorsal horn and that neuronal *Gabrg1* expression is more prevalent in the superficial than in the deep dorsal horn.

### Mice lacking γ2 subunits from the spinal cord

**Figure 3.**
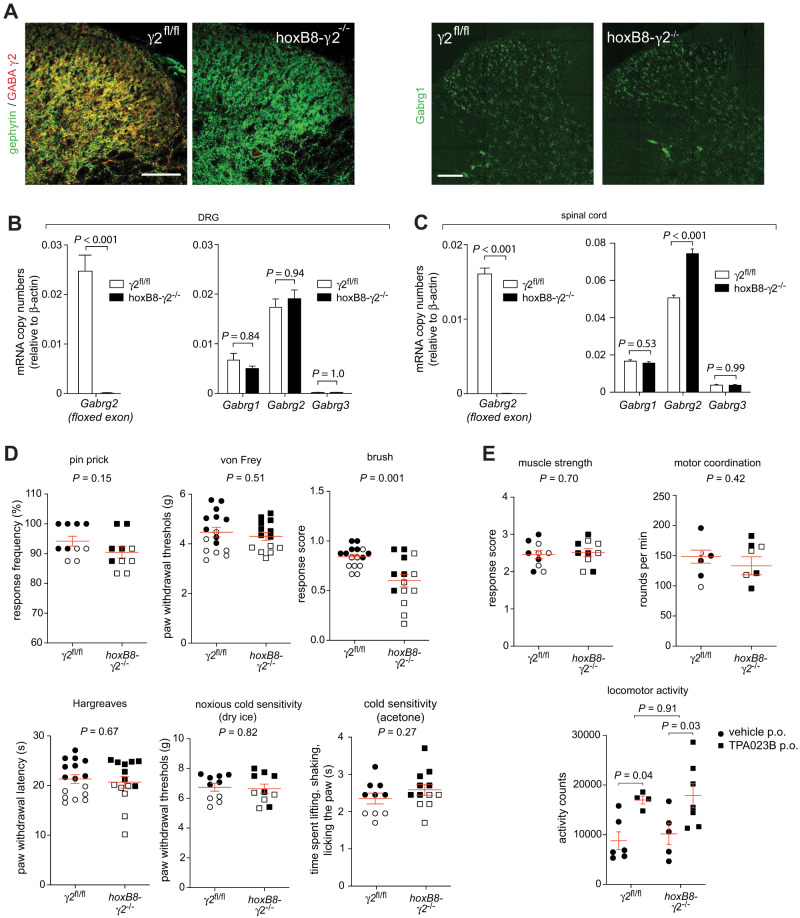
Nociceptive sensitivity of *hoxB8γ2*^−/−^ mice. ***A***, ***B***, Verification of the loss of γ2 subunit expression in *hoxB8γ2*^−/−^ mice and unchanged *Gabrg1* expression in *hoxB8γ2*^−/−^ mice. ***A***, Left, Transverse lumbar spinal cord sections of a *γ2*^fl/fl^ (left) and a *hoxB8γ2*^−/−^ mouse (right) stained for gephyrin and GABA_A_R γ2 subunit protein. Right, Comparison of *Gabrg1* expression (FISH) in *γ2*^fl/fl^ and *hoxB8γ2*^−/−^ mice. Scale bar, 100 µm. ***B***, Left, Quantification of *Gabrg2* mRNA relative to β-actin in lumbar DRGs of *γ2*^fl/fl^ (*n* = 4–6) and *hoxB8γ2*^−/−^ (*n* = 8–9) mice. *Gabrg2* mRNA was detected with a probe binding to the sequence flanked by the two loxP sites. Unpaired test. Right, Quantification of the GABA_A_R γ subunit expression in DRGs of *γ2*^fl/fl^ (*n* = 4–6) and *hoxB8γ2*^−/−^ mice (*n* = 6). Here, *Gabrg2* mRNA was detected with a probe binding a sequence outside the region flanked by the two loxP sites. *t* tests followed by Bonferroni’s correction for multiple testing. ***C***, Same as ***B***, but lumbar spinal cord tissue (*n* = 8, for both *γ2*^fl/fl^ and *hoxB8γ2*^−/−^ mice). ***D***, Nociceptive and somatic sensitivity. Mechanical sensitivity was tested in the pin prick, the von Frey test, and with a soft paint brush. Thermal sensitivity was assessed in the Hargreaves test, in the cold plantar test (dry ice) and the acetone test. Unpaired *t* tests, *n* = 10–14 and 10–16 for *hoxB8γ2*^−/−^ mice and *γ2*^fl/fl^ mice, respectively. Closed and open symbols indicate male and female mice, respectively. ***E***, Muscle strength, motor coordination, and effects of TPA023B (1 mg/kg, p.o.) on locomotor activity assessed in the horizontal wire test, the rotarod test, and the actimeter test, respectively. Muscle relaxation, unpaired *t* test, *n* = 10 and 10 for *hoxB8γ2*^−/−^ mice and *γ2*^fl/fl^ mice. Motor coordination, unpaired *t* test, *n* = 8 and 8 for *hoxB8γ2*^−/−^ mice and *γ2*^fl/fl^ mice. Locomotor activity. Two-way ANOVA followed by Bonferroni’s post hoc test. Treatment * genotype interaction *F*_(1,18)_ = 0.014, *n* = 6–7 and 4–6 for *hoxB8γ2*^−/−^ mice and *γ2*^fl/fl^ mice. Circles and squares represent individual mice. Closed and open symbols indicate male and female mice, respectively. Mean ± SEM.

To further investigate the function of GABA_A_R γ subunits, we decided to study the consequences of genetic ablation of γ2 GABA_A_R subunit. Since most mice that lack GABA_A_R γ2 subunits globally die early after birth (Essrich et al., 1998), we used a strategy that allowed us to delete GABA_A_R γ2 subunits from the spinal cord and DRG but to retain expression in the brain. To this end, we crossed mice carrying a floxed *Gabrg2* allele (Schweizer et al., 2003) with transgenic mice that express the Cre recombinase under the transcriptional control of the *hoxB8* gene (Witschi et al., 2010, see also Paul et al., 2014). *HoxB8-γ2*^−/−^ mice were viable and showed no obvious anatomical or behavioral abnormalities. Using immunocytochemistry and qRT-PCR, we verified that γ2 GABA_A_Rs were completely absent from the spinal cord and DRG (Fig. 3*A–C*). FISH experiments demonstrate that the gross expression pattern of *Gabrg1* was not altered in dorsal horn sections taken from *hoxB8-γ2*^−/−^ mice (Fig. 3*A*, right panels). Accordingly, neither *Gabrg1* nor *Gabrg3* mRNA were altered in the DRG or spinal cords of *hoxB8-γ2*^−/−^ mice (Fig. 3*A*, left panel). We found, however, an upregulation of *Gabrg2* mRNA when probes were used that bind to mRNA outside the deleted region, indicating the presence of some homeostatic processes (Fig. 3*B*,*C*). The presence of *Gabrg2* mRNA in *hoxB8-γ2*^−/−^ mice raises the possibility that a truncated protein might have be expressed in the *hoxB8-γ2*^−/−^ mice. However, the γ2 antibody used in this study, which was raised against the N-terminal 29 amino acids of the γ2 GABA_A_R subunit (Fritschy and Mohler, 1995), did not detect any remaining γ2 GABA_A_R subunit protein in the *hoxB8-γ2*^−/−^ mice indicating that the remaining mRNA was not translated into protein (compare Fig. 3*A*). Furthermore, any remaining γ2 GABA_A_R subunit protein would lack the transmembrane segment (TM) 3, part of TM2, and part of the large intracellular loop (Günther et al., 1995) and would therefore be nonfunctional.

### Spinal cord-specific deletion of γ2 GABA_A_Rs does not alter nociceptive sensitivity

Loss of synaptic inhibition in the spinal dorsal horn, for example through blockade of spinal GABA_A_Rs, induces exaggerated nociceptive reactions (for a review, see Zeilhofer et al., 2012). We therefore tested whether spinal cord-specific deletion of spinal GABA_A_R γ2 subunits would alter the sensitivity of mice in a battery of sensory and nociceptive tests (Fig. 3*D*). Unexpectedly, sensitivity to noxious mechanical, heat, and cold stimuli was indistinguishable from that of wild-type (*γ2*^fl/fl^) mice. We also found no differences in muscle strength, assessed in the horizontal wire test, and in the rotarod test, a measure of motor coordination (Fig. 3*E*). The only significant difference discovered was a decreased responsiveness to light dynamic touch. Unaltered sensitivity to noxious stimuli suggests that synaptic inhibition was sufficiently retained in the superficial layers of the dorsal horn, where nociceptive signals are processed. The observed change in responsiveness to dynamic touch stimuli may reflect a change in synaptic inhibition in the deep dorsal horn, where signals from innocuous mechanical stimulation are processed. The decreased rather than increased sensitivity may suggest the presence of a disinhibitory circuit involving γ1 GABA_A_Rs expressed on GABAergic neurons. To verify that supraspinal γ2 GABA_A_Rs were intact, we tested the effect of TPA023B, an α2/α3 GABA_A_R subtype-selective BDZ site agonist, that increases spontaneous locomotion in mice probably via its anxiolytic activity (Ralvenius et al., 2018). No differences were found in TPA023B-induced increase in locomotion between *hoxB8-γ2*^−/−^ and *γ2*^fl/fl^ mice (Fig. 3*E*).

### GABA_A_R clusters in mice lacking γ2 subunits in the spinal cord

**Figure 4.**
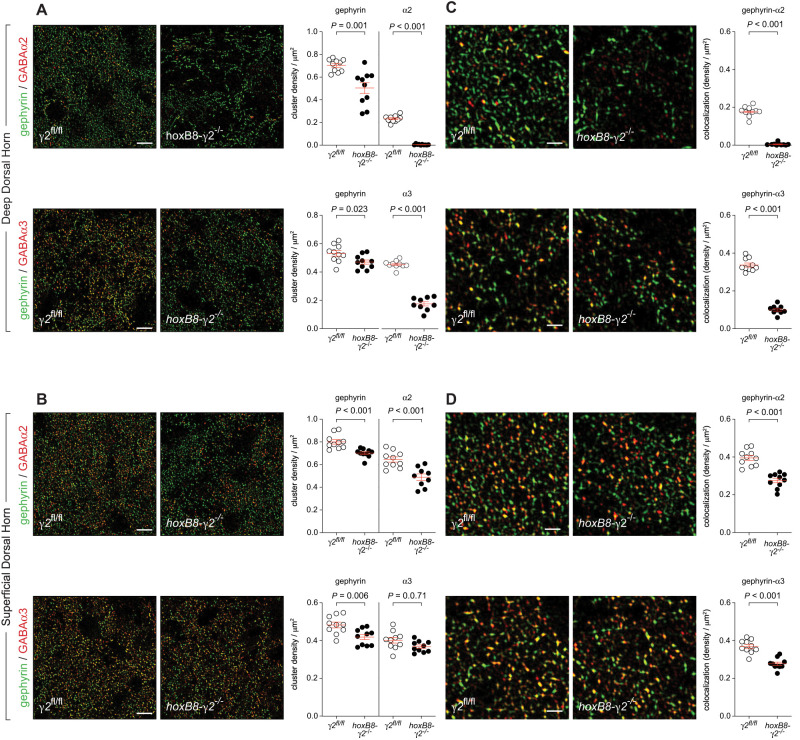
GABA_A_R clustering in the dorsal horn of *hoxB8γ2*^−/−^ mice. ***A***, Deep dorsal horn. Immunofluorescent staining of gephyrin (green) and GABA_A_R α2 subunits (top) or GABA_A_R α3 subunits (bottom) in *γ2*^fl/fl^ and *hoxB8γ2*^−/−^ mice. Statistics: Cluster density of gephyrin and GABA_A_R α2 and α3 subunits. Unpaired *t* tests. Mean ± SEM. ***B***, Same as ***A*** but superficial dorsal horn. ***C***, High-resolution images illustrating colocalization of gephyrin with GABA_A_R α2 and α3 subunits in the deep dorsal horn. Scale bars, 10 µm (left) and 3 µm (right). ***D***, Same as ***C*** but superficial dorsal horn. Individual dots represent one section. In total 9–10 sections from three mice were analyzed per condition.

To better understand why nociceptive responses remained unchanged despite the loss of γ2 GABA_A_R subunits in the spinal cord, we quantified postsynaptic GABA_A_R clusters in the dorsal horn of *γ2*^fl/fl^ and *hoxB8-γ2*^−/−^ mice (Fig. 4). We expected that the loss of γ2 would reduce the number of GABA_A_R clusters as γ2 subunits are essential for the association of GABA_A_Rs with the postsynaptic scaffold protein gephyrin (Essrich et al., 1998). To quantify GABA_A_R clusters, we stained transverse sections of lumbar spinal cord of *hoxb8-γ2*^−/−^ and *γ2*^fl/fl^ mice with antisera against α2 and α3 GABA_A_R subunits and against gephyrin. We started with analyses of the deep dorsal horn, where the γ1 GABA_A_R subunit is only weakly expressed. As expected, GABA_A_R clusters containing α2 or α3 GABA_A_R were almost completely absent or greatly diminished (α2 GABA_A_Rs: 0.23 ± 0.009/µm^2^ in *γ2*^fl/fl^ mice vs 0.005 ± 0.001/µm^2^ in *hoxb8-γ2*^−/−^ mice, equivalent to a reduction by 97.8%; α3 GABA_A_Rs: 0.46 ± 0.01/µm^2^ vs 0.18 ± 0.02, equivalent to a reduction by 60.9%; Fig. 4*A*). In contrast, in the superficial layers, where γ1 GABA_A_R subunits were more abundant, the numbers of α2 and α3 subunit containing GABA_A_R clusters were reduced to a lesser extent (α2 GABA_A_Rs: 0.64 ± 0.02/µm^2^ in *γ2*^fl/fl^ mice vs 0.50 ± 0.03/µm^2^ in *hoxb8-γ2*^−/−^ mice, equivalent to a reduction by 21.9%; α3 GABA_A_Rs: 0.40 ± 0.02/µm^2^ vs 0.37 ± 0.01, equivalent to a reduction by only 7.5%; Fig. 4*B*).

In most CNS areas, GABA_A_R cluster colocalizes with gephyrin. However, at certain sites, clustering apparently occurs in its absence (Kneussel et al., 2001; Levi et al., 2004; Panzanelli et al., 2011). We therefore analyzed whether the loss or retention of GABA_A_R clusters in the deep and superficial dorsal horn parallel with the changes in the number of GABA_A_R clusters containing gephyrin (Fig. 4*C*,*D*). We defined colocalization as points of spatial overlap between the signals generated by α2 or α3 GABA_A_R subunit markers with gephyrin markers. In the deep dorsal horn colocalization of gephyrin and α2 GABA_A_R subunits were virtually absent (0.003 ± 0.001 clusters/µm^2^ in *hoxb8-γ2*^−/−^ mice compared with 0.175 ± 0.004/µm^2^ in *γ2*^*f*l/fl^ mice, equivalent to a reduction by 92.3%), and colocalization between gephyrin and α3 GABA_A_Rs was reduced from 0.34 ± 0.01/µm^2^ in *γ2*^fl/fl^ mice to 0.10 ± 0.01/µm^2^ in *hoxb8-γ2*^−/−^ mice, equivalent to a reduction by 70.6% (Fig. 4*C*). In contrast, in the superficial dorsal horn of *hoxb8-γ2*^−/−^ mice, colocalization was reduced only by 30.2 and 25.0% for α2 and α3 GABA_A_Rs, respectively. These results demonstrate that the reduction in the number of GABA_A_R α2 and α3 clusters parallels the reduction of clusters containing GABA_A_R α2 or α3 subunits together with gephyrin (Fig. 4*D*). They hence suggest that neuronal *Gabrg1* contributes to the retention of GABA_A_R clusters in the absence of γ2 subunits. The colocalization of α2 and α3 GABA_A_Rs with gephyrin in the superficial dorsal horn indicates in addition that these clusters resided on intrinsic dorsal horn neurons rather than on sensory nerve terminals, which mostly lack gephyrin (Lorenzo et al., 2014).

### Agonistic activity of HZ-166 at γ1 GABA_A_Rs and retained antihyperalgesia by HZ-166 in hoxB8-γ2^−/−^ mice

**Figure 5.**
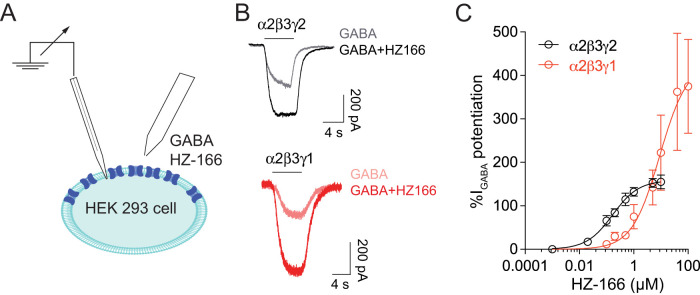
Activity of HZ-166 at γ1 and γ2GABA_A_Rs. ***A***, Schematic representation of the experiment. Potentiation of recombinant γ2 (α2β3γ2) and γ1 (α2β3γ1) GABA_A_R currents by HZ-166 was assessed in HEK 293 cells. GABA concentration was EC_5_ (1 μM for α2β3 γ2 and 10 μM for α2β3γ1). ***B***, Example trances of GABA evoked membrane currents in the presence and absence of a saturating concentration of HZ-166 (10 and 100 µM for γ2 and γ1 containing GABA_A_Rs. ***C***, Concentration response curve fitted to the Hill equation with a baseline fixed to 0. Number of cells, *n* = 6–11. Data are mean ± SEM.

The GABA_A_R γ2 subunit not only mediates synaptic clustering of GABA_A_Rs but, together with an α subunit, also forms the BDZ binding site. The majority of tested BDZ site agonists potentiate only γ2 GABA_A_Rs (Ymer et al., 1990; Wafford et al., 1993; Baburin et al., 2008). Some BDZ agonists, such as diazepam, flunitrazepam, and triazolam also potentiate γ1 GABA_A_Rs although with considerably lower potencies (Khom et al., 2006; Atack et al., 2011). We therefore asked whether BDZ site agonists with activity at γ1 GABA_A_Rs would exert at least part of their antihyperalgesic action through the potentiation of γ1 GABA_A_Rs. To avoid confounding sedative effects in these in vivo experiments, we tested whether the nonsedative BDZ site agonist HZ-166 (Rivas et al., 2009) would potentiate γ1 GABA_A_Rs (Fig. 5). We have previously shown that HZ-166 reduces inflammatory and neuropathic hyperalgesia without inducing sedation at antihyperalgesic doses (Di Lio et al., 2011). To test whether HZ-166 potentiates γ1 GABA_A_Rs, we compared the GABA_A_R current potentiation by HZ-166 in HEK 293 cells transiently transfected with either α2, β3, and γ1 or with α2, β3, and γ2 subunits. HZ-166 potentiated both subtypes of GABA_A_Rs. γ2 GABA_A_Rs were potentiated with an EC_50_ of 0.15 ± 0.01 µM and an *E*_max_ of 162.7 ± 19.5%. γ1 GABA_A_Rs were potentiated with lower potency (EC_50_: 8.8 ± 2.3 µM) but higher efficacy (*E*_max_: 375 ± 108%; Fig. 5*B*,*C*).

**Figure 6.**
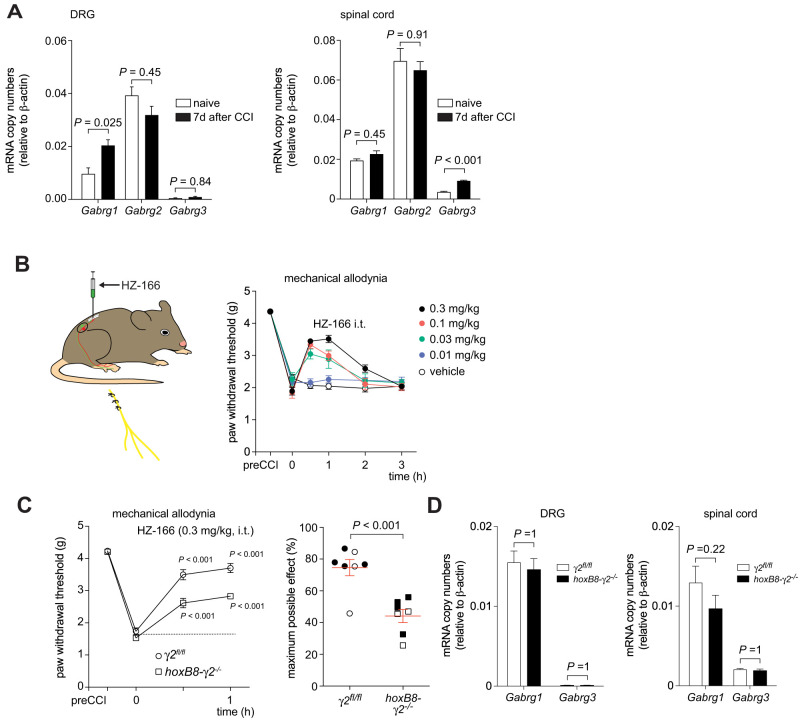
Antihyperalgesic actions of HZ-166 in mice with neuropathic hyperalgesia. ***A***, Changes in GABA_A_R γ subunit expression after peripheral nerve damage. qRT-PCR measurements of mRNA encoding for the three GABA_A_R γ subunits in lumbar DRG (left) and lumbar spinal cords (right), before and 7 d after CCI surgery (*n* = 8–10 mice per group). mRNA expression is expressed relative to β-actin expression. Statistics, DRG: ANOVA followed by Bonferroni’s post hoc test. *F*_(2,39)_ = 6.25. Spinal cord: *F*_(2,33)_ = 1.44. Error bars indicate mean ± SEM. ***B***, Dose-dependent reversal of mechanical hyperalgesia by HZ-166 7 d after CCI surgery. *n* = 9, 6, 7, 6, and 6 mice for vehicle, 0.01, 0.03, 0.1, and 0.3 mg/kg HZ-166 intrathecally. ***C***, Left panel (paw withdrawal threshold vs time), Partially retained antihyperalgesia by HZ-166 (0.3 mg/kg, i.t.) in hoxB8γ2^−/−^ mice. Repeated-measures ANOVA followed by Dunnett's post hoc test with predrug baseline as reference *F*_(2,12)_ = 73.5. Right panel (statistical analysis). Percent maximum possible effect averaged for time points 0.5 and 1 h. Unpaired *t* test. *n* = 7, for both *hoxB8γ2*^−/−^ mice and *γ2*^fl/fl^ mice, respectively. Closed and open symbols indicate male and female mice. ***D***, Expression of *Gabrg1* and *Gabrg3* in DRGs and spinal cords of *γ2*^fl/fl^ and *hoxB8γ2*^−/−^ mice 7 d after CCI surgery (mean ± SEM). *t* tests followed by Bonferroni’s correction for multiple testing. Data are mean ± SEM.

We then analyzed the antihyperalgesic effects of intrathecally injected HZ-166 in mice with neuropathic sensitization induced by a CCI surgery of the sciatic nerve (Fig. 6). Before starting with these behavioral experiments, we analyzed whether the expression of any of the γ GABA_A_R subunit would change in response to peripheral nerve injury. We found a significant upregulation of *Gabrg1* transcript numbers in DRG (0.0097 ± 0.0022 vs 0.020 ± 0.002; pre- vs post-CCI surgery; *t* test; *p *= 0.025 corrected for three independent tests) and a trend toward reduced expression of *Gabrg2*. *Gabrg3* remained at very low levels*.* In the spinal cord, we detected a significant upregulation of *Gabrg3* (0.0036 ± 0.0004 vs 0.092 ± 0.0003; pre- vs post-CCI surgery; *t* test; *p* < 0.001 corrected for threes independent test), but its expression level remained well below that of *Gabrg1* and *Gabrg2* (Fig. 6*A*). For pharmacological analyses in neuropathic mice, we chose an intrathecal delivery route to further rule out confounding effects resulting from supraspinal sites. In *γ2*^fl/fl^ mice, HZ-166 exerted pronounced dose-dependent antihyperalgesia (Fig. 6*B*), as previously reported (Di Lio et al., 2011). We next compared the antihyperalgesic effects obtained with HZ-166 at a dose of 0.3 mg/kg with those in *hoxB8-γ2*^−/−^ mice. HZ-166 was still antihyperalgesic in *hoxB8-γ2*^−/−^ mice albeit with reduced efficacy (Fig. 6*C*). In additional qRT-PCR experiments, we ruled out that the deletion of the γ2 subunit might have led to a differential regulation of *Gabrg1* or *Gabrg3* in DRG or spinal cords of mice after CCI surgery (Fig. 6*D*). GABA_A_R independent off-target effects of HZ-166 through receptors different from GABA_A_Rs can also be excluded since the antihyperalgesic effect of HZ-166 is absent from mice carrying BDZ-insensitive α2 GABA_A_Rs (Ralvenius et al., 2015). This result suggests that in addition to γ2 GABA_A_Rs, γ1 GABA_A_Rs contribute to HZ-166-induced antihyperalgesia.

## Discussion

In the present study, we investigated the potential role of γ1 GABA_A_Rs in spinal nociceptive processing. We studied the expression of the different GABA_A_R γ subunits in the spinal cord and DRG, and the impact of spinal cord-specific deletion of the γ2 GABA_A_Rs on the clustering of GABA_A_Rs and on nociceptive behavior. We also identified a compound with high efficacy at γ1 GABA_A_Rs, which shows antihyperalgesic effects in neuropathic mice lacking γ2 GABA_A_R from the spinal cord. Our results thus suggest a significant contribution of γ1 GABA_A_Rs to spinal nociceptive control.

### Distribution of γ1 GABA_A_Rs in the mouse CNS

The expression of γ1 GABA_A_Rs in the rodent brain has been analyzed previously. In most brain regions, its expression is negligible compared with that of γ2 GABA_A_Rs (Hortnagl et al., 2013). However, some areas, such as the caudate putamen, the colliculi, and the hippocampal complex, express low levels of *Gabrg1* mRNA, and in other areas, such as the amygdaloid and hypothalamic nuclei, *Gabrg1* mRNA expression appears even higher than that of *Gabrg2* (Ymer et al., 1990). Immunohistochemical studies have largely confirmed these results (Hortnagl et al., 2013). Expression of γ1 GABA_A_Rs in the spinal cord has not yet been reported in scientific articles, but the Gensat website reports expression in the superficial dorsal horn of adult mice (www.gensat.org/imagenavigator.jsp?imageID=12994), consistent with our results.

### Involvement of the γ1 subunit in GABA_A_R clustering

GABA_A_Rs cluster at postsynaptic membranes via an interaction of the γ subunit with the scaffolding protein gephyrin. For the vast majority of GABA_A_Rs and CNS areas, this occurs via the γ2 subunit (Essrich et al., 1998). During prenatal development, expression of the γ3 subunit is largely delimited to the developing forebrain where it can contribute to BZD modulation of postsynaptic GABA_A_Rs upon deletion of the γ2 subunit (Baer et al., 1999). In our experiments, deletion of the γ2 subunit from the spinal cord had contrasting effects in the superficial and deep dorsal horn, with nearly abolished clustering in the deep dorsal horn and only minor reductions in cluster numbers in the superficial dorsal horn. The majority of clusters retained in the superficial dorsal horn contained besides GABA_A_R α subunits also gephyrin, indicating that they resided on intrinsic dorsal horn neurons rather than on sensory afferent terminals (Lorenzo et al., 2014). This difference between the superficial and deep dorsal horn correlates with the abundance of the γ1 subunit and suggests that these clusters were formed via an association of gephyrin with the γ1 subunit. This is consistent with previous reports showing that γ subunits different from γ2 can also support clustering (Baer et al., 1999; Dixon et al., 2017).

### Role of γ1 GABA_A_Rs in the spinal control of nociception

The presence of the γ1 subunit in the superficial dorsal horn suggests that γ1 GABA_A_Rs contribute to the processing of nociceptive signals. At sites, where most GABA_A_Rs contain the γ2 subunit, its deletion should not only reduce the number of GABA_A_Rs but also affect its clustering at postsynaptic sites and hence strongly reduce the inhibitory tone. A loss of inhibitory tone in the dorsal horn, for example, through blockade of GABA_A_Rs with bicuculline, leads to strongly exaggerated nociceptive responses (Roberts et al., 1986). Such hyperalgesia was however not observed in the *hoxb8-γ2*^−/−^ mice investigated in the present study. As our mRNA expression analyses indicate, the absence of a nociceptive phenotype did not result from a compensatory upregulation of γ1 or γ3 GABA_A_R subunits suggesting that GABA_A_R clusters containing γ1 GABA_A_Rs were able to maintain sufficient synaptic inhibition.

The results of the colocalization experiments demonstrate that γ1 GABA_A_R subunits are coexpressed in superficial dorsal horn neurons with α2 and α3 GABA_A_R subunits, suggesting that they integrate into the GABA_A_Rs that mediate the antihyperalgesic and antipruritic effects of α2/α3 subtype-selective compounds, such as TPA023B (Ralvenius et al., 2018; Neumann et al., 2021). The partially retained antihyperalgesic effect of HZ-166 in *hoxB8-γ2*^−/−^ mice supports this idea. Finally, the proposed contribution of γ1 GABA_A_Rs to spinal nociceptive control is also in line with the results of a recent human genetics study, which discovered mutations in the coding region of the *GABRG1* gene in humans, and increased tactile sensitivity in point-mutated mice carrying one of these mutations in their genome (Dong et al., 2020).

Antihyperalgesia by HZ-166 very likely originates from an interaction with spinal GABA_A_Rs because HZ-166 was injected locally into lumbar intrathecal space and because previous work has demonstrated that the antihyperalgesic action of systemically applied HZ-166 originates from spinal rather than from supraspinal sites (Paul et al., 2014). The present results can however not differentiate between γ1 GABA_A_Rs residing on intrinsic dorsal horn neurons or on sensory axon terminals, and previous work has shown that both populations of receptors contribute about equally to BDZ site agonist-induced antihyperalgesia (Witschi et al., 2011). Furthermore, our FISH experiments revealed that more than half of the dorsal horn *Gabrg1* transcripts were localized in non-neuronal cells, i.e., in astrocytes, oligodendrocytes and microglia. Since all three glia types possibly contribute to chronic pain (Donnelly et al., 2020), these non-neuronal γ1 GABA_A_Rs may also contribute to the antihyperalgesic action of HZ-166 observed in our experiments.

### Pharmacological implications

Previous work has shown that positive allosteric modulators of spinal GABA_A_Rs reduce neuropathic and inflammatory hyperalgesia (Zeilhofer et al., 2015). Required doses of classical BDZ site agonists, including diazepam, are significantly higher than those inducing strong sedation (Ralvenius et al., 2015). Potentially clinically useful antihyperalgesia can therefore only be achieved with nonsedating α2 and α3 GABA_A_R subtype-selective (“α1 sparing”) compounds. Such compounds include, for example, L-838’417 (McKernan et al., 2000; Knabl et al., 2008), TPA023B (Atack, 2011; Ralvenius et al., 2018; Neumann et al., 2021) and HZ-166 (Rivas et al., 2009; Di Lio et al., 2011).

In the present study, we provide evidence for an antihyperalgesic effect of HZ-166, which occurs independent of γ2 GABA_A_Rs and which is most likely mediated by γ1 GABA_A_Rs. It is very well possible that the antihyperalgesic actions of other BDZ site agonists, including the approved drugs diazepam (Knabl et al., 2008; Ralvenius et al., 2015), (*N*-desmethyl) clobazam (Ralvenius et al., 2016), and the experimental compound TPA023B (Atack et al., 2011; Ralvenius et al., 2018; Neumann et al., 2021), partially originate from their interaction with spinal γ1 GABA_A_Rs.

Most BDZ site agonists have negligible activity and affinity at γ1 GABA_A_Rs. Some BDZ site ligands however such as diazepam, clonazepam, flunitrazepam, and triazolam bind and modulate γ1 GABA_A_Rs, albeit with much lower affinity than γ2 GABA_A_Rs (Khom et al., 2006). Some inverse BDZ site agonists (negative allosteric modulators) at γ2 GABA_A_Rs, such as DMCM and β-CCM, behave as BDZ site agonists (positive allosteric modulators) at γ1 GABA_A_Rs (Puia et al., 1991; Wafford et al., 1993). This feature may explain the paradox that not only BDZ site agonists but also inverse agonists exert antihyperalgesic activity (Sieve et al., 2001; Munro et al., 2011). The competitive BDZ site antagonist flumazenil (Ro 15-1788), which is often used as a radioligand of GABA_A_Rs (Herde et al., 2017), loses its affinity at GABA_A_Rs when the γ2 subunit is replaced by γ1 (McKernan et al., 1995). These effects suggest the presence of structural differences in the BDZ binding site of γ2 and γ1 subunits. Indeed, the phenylalanine (77F) residue at position 77 in γ2 GABA_A_R subunit, which is critically involved in the binding of classical BDZ site agonists (Cope et al., 2004), is replaced by an isoleucine (I) at the corresponding site of the γ1 GABA_A_R subunit. Such structural differences may offer an opportunity for the development of γ1 GABA_A_R-specific BDZ site ligands.

### Conclusion

In summary, our results suggest that γ1 GABA_A_Rs are present in the superficial layers of the dorsal horn in physiologically and pharmacologically relevant amounts. They contribute to the spinal control of nociception and likely mediate part of the antihyperalgesic effects of BDZ site agonists with activity at γ1 GABA_A_Rs. Since γ1 GABA_A_Rs constitute only a small portion of GABA_A_Rs in most parts of the CNS, specific targeting of these receptors may offer an additional path to better tolerated BDZ site ligands.
